# Adverse drug reactions in older adults: a narrative review of the literature

**DOI:** 10.1007/s41999-021-00481-9

**Published:** 2021-03-18

**Authors:** Maria Beatrice Zazzara, Katie Palmer, Davide Liborio Vetrano, Angelo Carfì, Graziano Onder

**Affiliations:** 1 Department of Gerontology, Fondazione Policlinico Gemelli IRCCS, Rome, Italy; 2 Aging Research Center, Department of Neurobiology, Care Sciences and Society, Karolinska Institutet, Stockholm, Sweden; 3 Department of Cardiovascular, Endocrine-Metabolic Diseases and Aging, Istituto Superiore di Sanità, Rome, Italy

**Keywords:** Older adults, Adverse drug reactions, Multimorbidity, Frailty, Polypharmacy

## Abstract

**Purpose:**

Adverse drug reactions (ADRs) represent a common and potentially preventable cause of unplanned hospitalization, increasing morbidity, mortality, and healthcare costs. We aimed to review the classification and occurrence of ADRs in the older population, discuss the role of age as a risk factor, and identify interventions to prevent ADRs.

**Methods:**

We performed a narrative scoping review of the literature to assess classification, occurrence, factors affecting ADRs, and possible strategies to identify and prevent ADRs.

**Results:**

Adverse drug reactions (ADRs) are often classified as Type A and Type B reactions, based on dose and effect of the drugs and fatality of the reaction. More recently, other approaches have been proposed (i.e. Dose, Time and Susceptibility (DoTS) and EIDOS classifications). The frequency of ADRs varies depending on definitions, characteristics of the studied population, and settings. Their occurrence is often ascribed to commonly used drugs, including anticoagulants, antiplatelet agents, digoxin, insulin, and non-steroidal anti-inflammatory drugs. Age-related factors—changes in pharmacokinetics, multimorbidity, polypharmacy, and frailty—have been related to ADRs. Different approaches (i.e. medication review, software identifying potentially inappropriate prescription and drug interactions) have been suggested to prevent ADRs and proven to improve the quality of prescribing. However, consistent evidence on their effectiveness is still lacking. Few studies suggest that a comprehensive geriatric assessment, aimed at identifying individual risk factors, patients’ needs, treatment priorities, and strategies for therapy optimization, is key for reducing ADRs.

**Conclusions:**

Adverse drug reactions (ADRs) are a relevant health burden. The medical complexity that characterizes older patients requires a holistic approach to reduce the burden of ADRs in this population.

## Key summary points

**Aim:**

To summarize the classification and occurrence of ADRs and identify risk factors and strategies to reduce and prevent ADRs in older adults.

**Findings:**

In frail, multimorbid older adults, who are often treated with polypharmacy, ADRs are frequently associated with health burden and hospitalization. Multiple age-related risk factors, including changes in pharmacokinetics, multimorbidity, polypharmacy, and frailty can increase the risk of ADRs, and different strategies have been suggested to prevent the onset of ADRs.

**Message:**

A multidimensional and holistic approach combining pharmaceutical interventions with a global evaluation of health needs and priorities can reduce the burden of ADRs in older adults.

## Introduction

Adverse drug reactions (ADRs) are defined as any noxious, undesired, or unintended response to a therapeutic agent, which may be expected or unexpected, and may occur at dosages used for the prophylaxis, diagnosis, or therapy of disease, or for modifying physiological function. ADRs do not include therapeutic failures, poisoning, accidental or intentional overdoses [1]. ADRs are common in clinical practice and they often represent the cause of unplanned hospitalizations [2], particularly in older adults, who frequently receive multiple drugs and often present with multiple conditions (multimorbidity) [3]. ADRs are considered a health priority since they are often preventable but can have a substantial impact on health outcomes and increase health care costs [4].

In this study, we performed a narrative scoping review of the literature to assess the impact of ADRs in older adults.

We completed a computerized literature search of relevant articles written in English with the aim of assessing the classification and occurrence of ADRs in the older population, evaluate the role of age and other risk factors for ADRs, and identified possible interventions to prevent the onset of this condition. References of interest were identified through searches of Pubmed and Google Scholar. Combinations of search terms were “adverse drug reactions”, “polypharmacy”, “multimorbidity” and “adverse drug reactions in older adult”, and the search terms were used alone or in combination. The reference lists of original articles and systematic reviews were hand-searched for other relevant articles.

## Classification

Different methods can be used to classify ADRs [4–6]. The first classification, suggested by Thomson and Rawlins 1981, classifies ADR into Type A and Type B reactions. Type A reactions occur in response to drugs given at therapeutic dose and are the result of an abnormal response of an otherwise normal pharmacological effect of a certain medicine. They are common but unlikely to be associated with a fatal event. On the other hand, Type B reactions are unrelated to the pharmacological effect or the dosage of the drug and are often fatal. This classification, as shown in Table 1, has been further updated with the inclusion of four other types of reactions: Type C reactions, related to the cumulative dose of a long-term pharmacological therapy; Type D reactions, related to the timing of a treatment; Type E reactions, related to the withdrawal of a given medicine; and Type F reactions, occurring when a therapy fails to be effective [4, 6].

**Table 1:** Classifications of adverse drug reactions

Type of reaction	Type of effect	Characteristics	Frequency	Examples	Management
A	Augmented	Dose related Low mortality Predictable	Common	Orthostatic hypotension with antihypertensive medications Respiratory depression with opioids Bleeding with warfarin; serotonin Syndrome with SSRIs Digoxin toxicity Anticholinergic effects of tricyclic antidepressants	Dose reduction Withdrawal drug if necessary Evaluation of effects of concomitant therapy and drugs’ interaction
B	Bizarre	Non-dose related High mortality Unpredictable	Uncommon	Hypersensitivity reactions such as anaphylaxis to penicillin Idiosyncratic reactions such as malignant hyperthermia with anaesthetics	Mandatory withdrawal of the drug Avoidance of that same drug in the future
C	Chronic	Cumulative Dose related Time related	Uncommon	Hypothalamic–pituitary–adrenal axis suppression by corticosteroids	Dose reduction Withdrawal drug if necessary, often for a prolonged period of time
D	Delayed	Often dose related Time related + +	Uncommon	Tardive dyskinesia Teratogenesis Carcinogenesis	Often non-treatable
E	End-of-treatment	Related to withdrawal time	Uncommon	Myocardial ischaemia after β-blocker discontinuation; Withdrawal syndrome with opiates or benzodiazepines	Slow withdrawal Reintroduction of the drug
F	Unexpected failure of therapy	Dose related Drugs’ interactions related	Common	Resistance to antimicrobial agents Inadequate dosage of an oral contraceptive if used with an enzyme inducer	Increase of dosage Evaluation of effects of concomitant therapy and drugs’ interaction

**Fig. 1:**
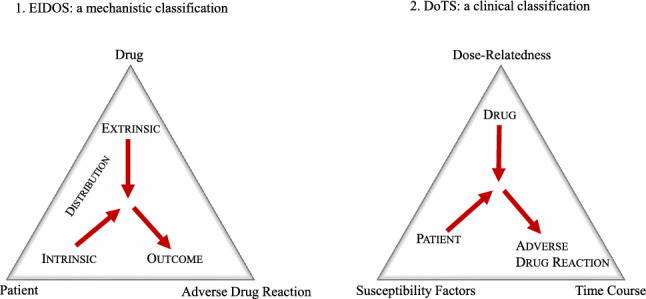
Comparison of dose, time and susceptibility DoTS and EIDOS adverse drug reaction classifications

Alternative classifications are represented by the Dose, Time and Susceptibility (DoTS) classification and the EIDOS scheme (Fig. 1). The first takes into account the dose of the drug, the time within which the reaction has occurred, and whether intrinsic susceptibility factors have contributed to the reaction [2, 7]. The DoTS classification describes clinical aspects of the reactions and is helpful in pharmacovigilance and identifying new adverse reactions in clinical settings. The EIDOS classification takes into consideration Extrinsic chemical species (E) supposed to initiate the effect; the Intrinsic chemical species (I) involved; the Distribution (D) of these species in the body; the Outcome (O) and the Sequela (S), which is the final adverse drug reaction [8]. The EIDOS classification analyses the biochemical mechanisms behind the adverse reactions and whether they could be caused by the molecule itself or a contaminant or an excipient or if there could be individual alterations in the distribution volume or individual differences in receptors’ actions. These two classifications, by analysing different aspects of ADRs, are complementary, adding different aspects so that, if used together, can help to comprehensively define and address ADRs [8].

In addition, it is important to classify the causal link between an observed ADR and a suspected drug. Due to the variety of manifestations, ADRs can be misinterpreted as symptoms or signs of a pathological state, rather than effects of medications. An ADR may present as a cardiovascular condition (i.e. syncope) or non-cardiovascular condition such as falls or gastrointestinal bleeding [9]. When assessing a patient’s medication history, especially in patients with advanced age, clinicians should be cautious to detect a possible connection between a clinical manifestation and a specific drug. Naranjo et al. developed an ADR Probability Scale which can be a useful tool to assess and classify the causal link between the ADR and the suspected drug [10]. The scale is composed of 10 items and can be quickly completed in a clinical setting. The overall score gives a probability that the adverse event is related to a drug reaction [10].

## Occurrence

The occurrence of ADR varies according to the strategy used to define and detect this condition, by characteristics of the studied population, and by the study setting. Most of the available studies focus on hospital settings as hospitalized patients can be closely monitored for the occurrence of ADRs. In addition, they are usually frail and present with acute diseases, which may further increase the number of prescribed drugs, and susceptibility to adverse medication effects, while raising the severity of drug-related illnesses. These factors make hospitalized populations an important target to study the occurrence of ADRs.

The European Commission has estimated that approximately 5% of all hospital admissions are due to ADRs and 5% of hospitalized patients will experience an ADR during their hospital stay. In 2008, in Europe, 197,000 deaths per year were attributed to ADRs [11]. A more recent exploratory review by Bouvy et al. assessed 32 studies from different settings and 12 different countries. This review included prospective and retrospective observational studies that evaluated the occurrence of ADRs by measuring the number of hospitalizations caused by ADRs, number of ADRs during hospitalization, and number of ADRs in outpatient settings in a specific period of time. The analysis of the studies included in the review showed an overall ADR rate of 3.6% at hospital admission and 10.1% during the hospital stay. Only five studies assessed the occurrence of ADR in community-dwelling older adults and reporting largely variable estimates. The overall proportion of fatal ADRs was found to be approximately 0.5% [12] and Type A reactions were the most common type of ADRs.

In the United States (US), Budnitz et al. [13] estimated that, among all visits to the emergency department in 2004 and 2005, more than 700,000 US patients were admitted due to adverse drug events and 3,487 were hospitalized. Hospitalization was used as a measure of the severity of the event [13]. In this case, older patients (≥ 65 years) accounted for 25.3% of emergency department visits attributed to adverse drug-related events (ADEs) and 48.9% of events requiring hospitalization. Patients aged 65 years and older were estimated to be twice more likely to have ADEs than younger patients (rate ratio (RR), 2.4; 95% confidence interval (CI), 1.8–3.0) and 7 times more likely to be hospitalized (RR 6.8; 95% CI 4.3–9.2) [13]. Warfarin, insulin, and digoxin were found to be the causative agents for almost a third of the admissions in patients aged 65 years or older [13]. Antibiotics were also frequently the cause of the ADEs, representing 1/8 of the estimated events treated in the emergency department (13.0%; 95% CI 11.7–13.3%) [13].

In another work by the same group, which focused specifically on hospitalisations caused by ADEs in adults aged ≥ 65 years, the authors observed that nearly half the hospitalizations were among adults 80 years of age or older (48.1%; 95% CI 44.6–51.6) and two-thirds of these were due to unintentional overdoses (65.7%; 95% CI 60.1–71.3) [14]. Overall, 67.0% (95% CI 60.0–74.1) of hospitalizations were attributed to few commonly used medications/class medications: warfarin (33.3%), insulins (13.9%), oral antiplatelet agents (13.3%), and oral hypoglycemic agents (10.7%) [14].

A systematic review examined drugs most commonly responsible for ADRs [15]. This study found that 51% of preventable drug-related hospital admissions were associated with only four groups of drugs. In particular, antiplatelet agents (16%), diuretics (16%), non-steroidal anti-inflammatory drugs (NSAIDs) (11%), and anticoagulants (8%) accounted for the majority of the events, supporting the idea that ADRs are highly associated with frequently used medications. Moreover, preventable ADRs were found to be commonly related to prescribing problems, low adherence to treatment, and insufficient monitoring of medications, highlighting how many ADRs could have been avoided by optimizing care planning and management.

Few studies have examined the occurrence of ADRs in the long-term care sector. A prospective cohort study of long-term care residents in USA found that at least 14% presented an ADR over a 12-month period [15]. In a study addressing ADR-related hospitalizations in nursing home residents, 15.7% of the 332 residents had at least one hospitalization and this event was found to be directly related to the number of medications taken per day. The medications most frequently involved were NSAIDs, psychotropic drugs, digoxin, and insulin [16]. Similarly, in a more recent study in American nursing home residents, antipsychotics [odds ratio (OR) 3.4; 95% CI 1.2–5.9), anticoagulants (OR 2.8; 95% CI, 1.6–4.7)], diuretics (OR 2.2; 95% CI 1.2–4), and anti-epileptic medications (OR 2.0; 95% CI 1.1–3.7) were found to increase the risk of a preventable adverse event [17] and the most common manifestations were delirium, over-sedation, and falls [17, 18].

Finally, the occurrence of ADRs in older adults might be underestimated due to a high rate of under-reporting and early detection of a new sign or symptom as an ADRs might be undermined. A systematic review by Hazell and Shakir, evaluating 37 studies from 12 different countries, documented a median under-reporting rate of 94% (interquartile range 82–98%). Despite demonstrating a higher evidence of under-reporting in general practice, the authors found no significant difference between hospital-based studies and general practice. Severe ADRs were less likely to be under-reported in hospital settings [19].

## Older age as a risk factor for ADRs

Several studies suggest that older adults can present with a higher rate of ADRs compared to younger adults and that increasing age can represent a risk factor for the occurrence of ADRs. For this reason, Stevenson et al. recently suggested that a broad approach is needed to address ADRs in the older population and that drug-related harm should be treated as a geriatric syndrome itself [20]. Several factors associated with increasing age can have a role in increasing the risk of ADRs.

### Drug metabolism changes

Ageing affects homeostasis and is related to physiological changes and conditions which are likely to increase the risk of iatrogenic events [21, 22]. Age-related changes in pharmacokinetics, and conditions such as multimorbidity, frailty, and polypharmacy (long-term use of ≥ 5 medications) can play a crucial role in this phenomenon [3, 13, 14, 23]. Alterations in pharmacokinetics affect drug metabolism and clearance [24] and increase the risk of ADRs or drug responsiveness. Alterations in volumes of drug distributions, due to decrease in total body water and different body fat distribution, can contribute to prolonging the half-life of a certain drug incrementing the risk of toxicity [3, 22]. Drug metabolism in patients on polypharmacy can also be affected by drugs–cytochrome P450 (CYP) interactions. Across-sectional study in a sample of institutionalized and community-dwelling octogenarians demonstrated that 72.2% of recruited participants presented a potential CYP drug–drug interaction, which influenced not only functional capacity and mobility, but also their self-perceived health status [25]. Aging also affects sex steroid hormone levels which have been found to determine sex differences in adverse response to medications, with women being more susceptible to ADRs [26, 27]. In particular, sex hormones may alter the pharmacokinetics of drugs by competing for their blood transporter or enzyme [28].

### Frailty

The accumulation of biological deficits and dysfunctions that characterize the aging process [29] may ultimately lead to frailty [30]. Frailty, as well as the above-mentioned physiological changes, can have a significant impact on the development of possible ADRs. Cullinan et al. [31] evaluated 711 patients with a frailty index (FI) ranging from 0 to 0.51 (mean 0.15) showing that patients with a FI ≥ 0.16 were twice as likely to develop at least one ADR during hospitalization and to experience a potentially inappropriate prescription, as defined by the Screening Tool of Older Person’s Prescriptions (STOPP) criteria [32], demonstrating a significant correlation between frailty and ADRs and inappropriate prescription [31].

### Multimorbidity

Multimorbidity is defined as the concomitant presence of two or more coexisting chronic diseases in the same individual [33] and is a major issue in geriatrics because its prevalence increases with age. Multimorbidity in older adults has a clear correlation with the occurrence of iatrogenic illness and several studies have suggested that the risk of ADRs increases with an increasing number of chronic diseases. This phenomenon could be caused by higher chances of drug-disease interaction—when a medicine used to treat one condition exacerbates the symptoms or signs of another underlying disorder—or the presence of a condition that can alter drugs’ metabolism, such as kidney and liver disease [21, 34]. Two classic examples of this phenomenon are beta-blockers taken for cardiovascular disease that can worsen asthma symptoms or metoclopramide for gastric dysmotility that can worsen motor symptoms in patients with Parkinson’s disease [21].

### Geriatric syndromes

Geriatrics syndromes, such as falls, delirium, cognitive impairment, orthostatic hypotension, incontinence, and chronic pain, may reduce the potential benefit of pharmacological treatment [21, 35–37], increase the risk of ADRs [21] and the rate of inappropriate prescriptions [38]. For example, patients can suffer from orthostatic hypotension and the use of antihypertensive medications can worsen this condition, leading to falls [21]. Similarly, older adults taking oral antidiabetics are more prone to hypoglycaemia, increasing the risk of falls [35]. Antiepileptics, antidepressants, and some antiparkinsonism drugs have been associated with an increased risk of delirium and incontinence [35]. Treatment for chronic pain, such as opioid agonists, has also been related to delirium and can increase the risk of falls [35]. Indirectly, some treatment can also have fatal consequences. For example, patients with atrial fibrillation at high risk of falls undergoing anticoagulant treatment demonstrated an increased risk of intracranial bleeding [39].

### Cognitive and sensory impairment

Conditions affecting cognition are also relevant in relation to possible patient errors or non-adherence to treatment plans. Cognitive impairment, mental illness, or simply poor vision are factors that are likely to increase the risk of errors and should be taken into account when prescribing. Functional deficits and cognitive impairment, characterized by memory loss, decline in intellectual function, impaired judgment and language, can pragmatically reduce the ability to manage pill containers and affect decision-making capacity. Therefore, cognitive impairment may impact not only the overall compliance but also result in underreporting of ADRs [21, 40]. In a study of 30,000 adults aged 65 years and over, 23.5% of all adverse drug events and 13.6% of potential adverse drug events were attributed to patient error. Errors occurred often in medication administration or self-modification of the treatment scheme [41]. Brauner et al. previously demonstrated that the use of medications to treat osteoporosis in people with dementia showed more risks than benefits to the patients, increasing the rate of developing serious iatrogenic illness [42].

### Polypharmacy

The most relevant age-related factor that could contribute to the higher prevalence of ADRs in older adults is polypharmacy. Older adults commonly use multiple drugs to treat multiple conditions. International estimates suggest that more than 60% of the older population receive five or more drugs concomitantly. The potential harm from drug reactions and interaction is increased by the higher number of medicines prescribed [36, 43] and the total number of drugs taken per day is an important risk factor for ADR-related hospitalizations [44]. It has been estimated that a person taking two medications has a 13% risk of experiencing an ADR; this risk increases to 58% and 82% when taking five and seven or more medications per day, respectively [3].

All the above-mentioned factors are summarized in Table 2. These factors partly explain the higher risk of ADRs observed in the older population and should be carefully considered when a new drug is prescribed. The majority of ADRs in older adults are Type A and they are predictable and preventable with adequate evaluation and monitoring [2, 3, 23]. Prudent prescribing is key to reducing errors and the risk of ADRs in the older population because it takes into account patient susceptibilities and medication history and considers non-pharmacological or conservative options [2]. Noticeably, older and frail people are often excluded from clinical trials assessing drug efficacy [29] and, therefore, reliably predicting the nature and incidence of adverse events in this population can be challenging [45]. Moreover, guidelines are often focused on the management of a single disease and rigidly relying on their guidance when prescribing can be detrimental when assessing older people with multimorbidity [3].

**Table 2:** Age-related factors associated with higher risk of ADRs in older adults

Factors	Possible mechanisms of action	Effect increasing the risk of ADR
Physiological age-related changes [21, 22, 24, 25, 28]	Changes in pharmacokinetics and pharmacodynamics of the drug	Alteration of drug metabolism and clearance
	Reduction in total percentage of body water Alterations in body fat distribution	Alteration in volume distribution of the drug Prolonged half-life of the drug
	Interaction with sex hormones transport/metabolism	Increased susceptibility to ADRs in women Competition of sex hormones for drug’s transporter or enzyme
Multimorbidity [21, 33, 34]	Drug–disease interaction	A drug given to treat a disease can worsen a co-existing disease
	Conditions altering drugs metabolism	Kidney and liver disease can alter drug metabolism
	Disorders determining non-metabolic reactions	Depression or other mental illness can amplify somatic symptoms with consequent higher report rate of ADRs
Polypharmacy [25, 36, 43, 44]	Drug–drug interactions	Additive/opposed pharmacological effect Pharmacokinetics and pharmacodynamics interactions between drugs causing treatment failure or toxicity
	Cytocrome P-450 interactions	Increased drug efficacy and toxicity
Frailty [29–32]	Increased vulnerability to stressors	Negative effects of drugs can be amplified
	Functional impairment (i.e. sight or hearing disability, walking difficulties)	Pragmatically reduced to manage pill containers Difficulties in reaching the pharmacy
Geriatric syndromes (i.e. delirium, falls, orthostatic hypotension)[35, 36, 38]	Continuation, recurrence or worsening of geriatric syndromes can be caused by drugs	Increased occurrence and severity of geriatric syndromes
Cognitive and sensory impairment [40–42]	Difficulties in managing therapy	Low adherence to treatment scheme Mistakes in taking medications

## Strategies to prevent ADRs in older adults

As the number of drugs received is one of the most relevant risk factors for ADRs, reducing drug burden can be considered as one of the most relevant interventions to reduce the risk of iatrogenic illness.

**Fig. 2:**
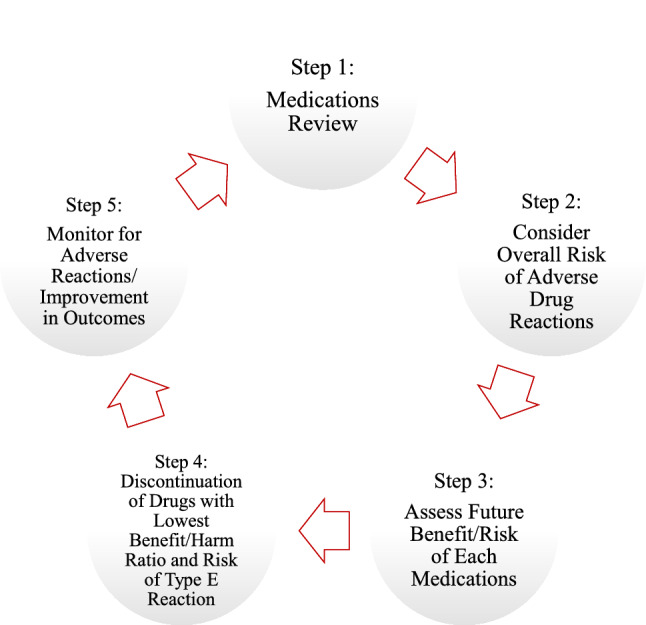
The deprescribing protocol: a five-step process by Scott et al. (2015)

Deprescribing is the process of withdrawing inappropriate medication or reducing posology under the supervision of a healthcare professional. The aim of deprescribing is to manage polypharmacy by reducing unnecessary or potentially harmful medication and improving outcomes [9, 46–48]. Scott et al. suggest a five-step protocol to facilitate the deprescribing process (Fig. 2) [48]. These steps include a systematic medicine revision to evaluate medication appropriateness based on the patient’s clinical state and overall functioning, life expectancy, and health priorities. Based on this knowledge, each medication should be carefully evaluated considering the risk of experiencing an ADR and the ratio risk/benefit for the patient. Once that the medicines to be discontinued are identified, monitoring for possible withdrawal reaction or improvements in outcome is fundamental [9, 48].

When deprescribing, clinicians should carefully prioritize overall benefit of a given drug, balancing the ratio risk/benefit [49]. For example, the Discontinuation of Antihypertensive Treatment in Elderly People (DANTE) study, which assessed the impact of deprescribing antihypertensive medications for 16 weeks in participants with mild cognitive impairment, reported no significant improvement in cognition nor an increase in adverse cardiovascular events when discontinuing antihypertensive drugs [50], supporting the advantage of deprescribing.

Both the prescribing and deprescribing process cannot take place without careful documentation of the patient’s health conditions. This includes the diagnosis of clinical and geriatric conditions, a thorough medication review (including herbal remedies or over-the-counter drugs), a precise analysis of possible previous ADRs, and a clear definition of health priorities and treatment goals [3]. In older people with polypharmacy, new drugs should be titrated slowly to reduce the risk of adverse events [3] and new symptoms should be considered as possible ADRs. This is fundamental for avoiding the possible activation of the prescribing cascade sequence. The prescribing cascade happens when an additional medication is prescribed to treat an ADR wrongly interpreted as a new medical condition [51]. A typical example of this process is the prescription of anti-Parkinson drugs to treat motor symptoms related to long-lasting antipsychotic therapy.

Other than adverse drug reactions, reasons for deprescribing are evident, for example, in the case of end of life or palliative care, where the most important goal is to treat symptoms and reduce treatment burden [9].

Several strategies or tools can support the deprescribing process:

### Drug review

Drug regimens should be periodically reviewed. The National Service Framework for Older People recommends that patients aged 75 years and over should have their treatment scheme reviewed at least once a year [41]. The aim of a medication review, which is a structured and critical examination of the person’s therapeutic plan, is to optimize the impact of the drugs and minimise the possibility of adverse reactions [52]. Moreover, to improve compliance, communication between different healthcare providers is highly advised [41], and some professionals, such as community pharmacists, can play a key role in the process of medication review. Community pharmacists can, in fact, help identify potential therapy-related problems and drive possible pharmaceutical interventions [53]. Deprescribing, avoidance of inappropriate prescribing—otherwise quite common in primary, secondary, and tertiary care [54–57]—and medicine reviews are important milestones to reduce the impact of ADRs in the older population.

### Tools to identify inappropriate prescribing

Several tools have been developed to facilitate the medication review process and foster deprescribing [58]. The American Geriatrics Society (AGS) Beer’s criteria [59] and the Screening Tool of Older Persons’ Potentially Inappropriate Prescriptions (STOPP) criteria [32] are commonly used. The STOPP criteria are often used in association with the Screening Tool to Alert doctors of Right Treatments (START) criteria that comprise 22 indicators of potentially important prescribing omissions in older people [32]. In an investigation of 4492 adverse drug events reported in 2004 and 2005, the Beer’s criteria medications were found to be associated with a fewer emergency department visits (3.6%) for ADRs in older adults as compared to other medications [60].

Similarly, the Fit fOR The Aged (FORTA) List represents a list of drugs that have been created via a consensus of experts with the aim of providing a validated clinical tool to increase the appropriateness of prescription and pharmacotherapy in older adults [61].

The FORTA lists label drugs chronically prescribed to older patients depending on safety, efficacy and age appropriateness. Drugs can be classified as A (A-bsolutely) when are indispensable, B (B-eneficial) when are certainly beneficial, C (C-areful) when their use is questionable, and D (D-on't) when the prescription of a given drug is definitely avoidable. Based on these categories, FORTA-labeled drug lists were approved in 7 European countries and U.S., reflecting the country-specific availability and usage of drugs and were lastly updated in 2018 [62–64]. In controlled clinical trials, the FORTA List demonstrated a significant impact on medication quality, reducing the risk of under and overtreatment mistakes and ADRs [64].

There are important differences between these tools with some just listing drugs to avoid, such as the Beer’s criteria, and some just labelling drugs appropriateness for a certain disease (i.e. FORTA list), overall failing to address a specific therapeutic situation or failing to provide special considerations of use and alternative therapies to avoid potentially inappropriate medications (PIMs) [65, 66]. Nevertheless, a recent systematic review concluded that none of the evaluated tools combine the various aspects of inappropriate prescribing, with each tool covering different aspects of medication review and management [67]. Another systematic review underlined how there was a wide variability between different PIMs’ lists, with little overlap between medications in different lists, and making it difficult for these tools to be applied in clinical practice [66].

### Software

Different computer software has been developed to provide support physicians at the time of the prescription and reduce ADRs and improve outcomes. The Clinical Decisions Support System (CDSS) and Computerized Prescription Support System (CPSS) are two software that works with different algorithms to identify potentially inappropriate prescriptions, risk of iatrogenic illness, appropriate drug dosage, drug interactions, and contraindicated treatments [68, 69]. Similarly, Computerized Provider Order Entry Systems (CPOE), is a system that allows healthcare providers to directly enter orders into a computer system and is able to detect and avoid possible errors [70]. However, very few studies were able to demonstrate an improvement in patient outcomes in relation to CPOE and CDSS usage [71] and there are several difficulties in addressing and managing ADRs. A randomized clinical trial showed how the use of CDSS was effective in reducing undesired drug–drug combination, but determined treatment delays if an immediate pharmacological therapy was needed, leading to the study being terminated early [72].

A meta-analysis by Grey et al. highlighted how different interventions are effective in reducing the risk of ADRs for 8 of the 15 study arms evaluated demonstrating an overall reduction in the number of serious adverse outcomes. Interventions differ greatly from study to study and most included pharmacist-led interventions or medication review in primary care. Only one study evaluated the impact of CDSS to assist pharmacists in identifying potential drug-related problems [73].

The Software ENgine for the Assessment & Optimization of drug and non-drug Therapy in Older peRsons (SENATOR) trial is a multinational randomised open-label blinded European Union-funded controlled trial started in 2012 and recently terminated in 2018 that aimed to ascertain the effect of the SENATOR software in optimizing medications prescriptions and non-pharmacological treatment in hospitalized older people with multimorbidity and polytherapy. By applying the STOPP and START criteria, the software produces a report which outlines possible drug–drug and drug–disease interactions and provides non-pharmacological recommendations aimed at reducing the risk of incident delirium. The primary endpoint of the study was to evaluate the percentage of patients with at least one probable or certain ADR occurring within 14 days of enrolment during the hospitalization period [74–76]. Unfortunately, the trial failed to show a significant impact in reducing the incidence of ADRs and the level of adherence by medical staff to the intervention was relatively low [77].

### Comprehensive geriatric assessment

A major limitation of the proposed approaches to reduce ADRs is that they focus mainly on pharmacological properties, undermining the complexity of older adults. These approaches have a limited consideration of the age-related factors that can increase the risk of ADRs, including frailty, multimorbidity, geriatric syndromes, and cognitive impairment. In addition, evaluation of patients’ preferences, health priorities, and life expectancy is rarely included in these interventions. For this reason, a global and comprehensive evaluation of patients’ needs could complement a “pharmaco-centric” approach in optimizing drug treatment and reducing ADRs. In this context, a large study of 834 frail older adults, evaluated the effect of a multidisciplinary and global approach based on Comprehensive Geriatric Approach and Management (CGAM) on ADRs. The authors demonstrated a 35% reduction in serious ADRs and inappropriate drug use [78] suggesting that CGAM combined with a systemic re-evaluation of the patient’s medication list is a fundamental tool for reducing ADRs [34]. In conclusion, by enabling the creation of multidimensional care plans for each patient, CGAM helps to avoid fragmented or poorly coordinated care and is a useful tool for defining treatment priorities and preventing ADRs in this population [3, 40].

## Conclusions

The medical complexity that characterizes older patients highlights the necessity of a holistic approach to this population. This is especially true when considering high-risk populations, such as long-term care facility residents or frail multimorbid hospitalized older adults [15]. Despite several tools having been developed to reduce the risk of ADRs, preventing ADRs is still very challenging. Reliance on guidelines for the management of single diseases is still quite common and often disadvantages older people with multimorbidity, increasing the risk of ADRs [3]. To reduce the burden of ADRs, approaches focused on pharmaceutical principles (i.e. medication review or software) should be addressed within the context of a global evaluation of patients’ characteristics, needs, and health priorities with the aim of tailoring prescriptions and care planning.
