# Correction to: Adverse drug reactions in older adults: a narrative review of the literature

**DOI:** 10.1007/s41999-021-00591-4

**Published:** 2021-11-22

**Authors:** Maria Beatrice Zazzara, Katie Palmer, Davide Liborio Vetrano, Angelo Carfì, Graziano Onder

**Affiliations:** 1 Department of Gerontology, Fondazione Policlinico Gemelli IRCCS, Rome, Italy; 2 Aging Research Center, Department of Neurobiology, Care Sciences and Society, Karolinska Institutet, Stockholm, Sweden; 3 Department of Cardiovascular, Endocrine-Metabolic Diseases and Aging, Istituto Superiore di Sanità, Rome, Italy

## Correction to: European Geriatric Medicine (2021) 12:463–473 10.1007/s41999-021-00481-9

In the original version of this article, the given name and family name of Graziano Onder were interchanged. The corrected author list is given above. The original article has been corrected.

